# Dr. Henry Gerhart

**Published:** 1901-03

**Authors:** 


### Obituary.

### DR. HENRY GERHART.

Dr. Gerhart died at his home in Lewisburg, Pa., on Sunday, January 20, 1901.

This simple announcement means more than that of the death of ordinary men, for Dr. Gerhart belonged to a class of which, unfortunately, there are too few, a class whose lives have broadened existence and made, unselfishly, paths for others to follow.

Dr. Gerhart was born at Mill Creek, Lancaster County, Pa., on April 8, 1827. He subsequently moved to Philadelphia and received his final school training in the High School of that city. After graduation his tastes led him into mechanics, and he entered the machine works at Eden, near Lancaster, Pa. At a later period he took up the study of dentistry and settled at Lewisburg in 1851, residing there to the date of his death.

Dr. Gerhart's work in dentistry in the State of Pennsylvania has been a most important one, but, like the influence of all earnest men, was felt in an ever-widening territory up to the period of his final departure from earthly labors, for at no time did he cease in his efforts for a higher standard of professional excellence.

He was one of the active organizers of the State Dental Society, and the first acquaintance the writer had with him was at the preliminary meeting that led up to this organization.

His intellectual force naturally made him a leader in whatever position he felt called upon to fill, and he was a frequent contributor of valuable papers to the literature of his profession. He was president of the State Society for several terms.

When the present law creating an examining board in dentistry went into effect he was appointed by Governor Hastings as one of its members, and was reappointed to that position by Governor Stone. It was through his high character, and that of the majority of the board, that helped materially to moderate the opposition felt by many to the re-examination of graduates.

The pastor of the Baptist church with which he had been connected for forty-eight years thus epitomized his deep reverential character for truth. "His admiration for the perfect character of Jesus and his matchless teachings was unbounded. In many things he agreed with the most pronounced type of advanced thinkers; in his search for truth he was a higher critic; in his philosophy of life he was an evolutionist, after the school of Charles Darwin; upon many subjects he was an agnostic; subjects upon which other men dogmatize with the easy egotism of ignorance were to him profound, unfathomed mysteries. . . . One doctrine he knew to be true,—the doctrine of God's infinite love."

Dr. Gerhart was active in educational matters. He became one of the trustees of Bucknell University in 1860, and served in that capacity for twenty-one years. The president of that University, in his remarks, said of him, "It was not in his official capacity that Dr. Gerhart most powerfully influenced the life of the various schools. It was by the power of his personality. His was a well-stored mind. His reading was wide and varied. His observation of men and things had been keen and extensive, and he was ready in the use of his varied fund of information. What he had read and observed he had made his own by reflection."

To satisfy this craving for knowledge of men and things he made extensive travels in Europe, the Holy Land, and Mexico; but it is said of him that he found so much of ignorance, greed, and bigotry wherever he went, that his travels were in the main disappointing.

He possessed great facility in the acquisition of languages, and made several of the modern languages equally his own.

When Dr. Gerhart died education lost a true friend and an enthusiastic devotee. Dentistry has reason to feel grateful that such a character was part of it through so many of the developing years of the nineteenth century, and that his splendid example is left us that we too may continue laboring for the high ideals that were the overmastering influence governing his life, and may so live and work that the future of our profession may be worthy of this man and of his highest conceptions.

Dr. Gerhart married Miss Susan Kennedy in 1851. His eldest son, Wilfred Gerhart, M.D., died in 1890. The other children were present at the funeral,—May, the wife of Rev. Joseph E. Perry, of Boston; Elizabeth, the wife of Mr. Edgar Dudley Faries, of Philadelphia; Mr. Rolf Gerhart, of Virginia, and Dr. Weber L. Gerhart, of Lewisburg.

